# Adding a second surprise question triggers general practitioners to increase the thoroughness of palliative care planning: results of a pilot RCT with cage vignettes

**DOI:** 10.1186/s12904-018-0312-6

**Published:** 2018-04-19

**Authors:** F. Weijers, C. Veldhoven, C. Verhagen, K. Vissers, Y. Engels

**Affiliations:** Department of Anesthesiology, Pain and Palliative Medicine, Radboud university medical center, Postbox 9101, internal code 549, 6500 HB Nijmegen, the Netherlands

**Keywords:** Palliative care, Surprise question, Identifying tool, General practitioner, RADIANT

## Abstract

**Background:**

In our aging society, palliative care should be a standard component of health care. However, currently it is only provided to a small proportion of patients, mostly to those with cancer, and restricted to the terminal phase. Many general practitioners (GPs) say that one of their most significant challenges is to assess the right moment to start anticipatory palliative care. The “Surprise Question” (SQ1: “*Would I be surprised if this patient were to die in the next 12 months”*?), if answered with “no”, is an easy tool to apply in identifying patients in need of palliative care. However, this tool has a low specificity. Therefore, the aim of our pilot study was to determine if adding a second, more specific “Surprise Question” (SQ2: *“Would I be surprised if this patient is still alive after 12 months*”?) in case SQ1 is answered in the negative, prompts GPs to plan for anticipatory palliative care.

**Methods:**

By randomization, 28 GPs in the south-eastern part of the Netherlands were allocated to three different groups. They all received a questionnaire with four vignettes, respectively representing patients with advanced organ failure (A), end stage cancer (B), frailty (C), and recently diagnosed cancer (D). GPs in the first group did not receive additional information, the second group received SQ1 after each vignette, and the third group received SQ1 and SQ2 after each vignette. We rated their answers based on essential components of palliative care (here called RADIANT score).

**Results:**

GPs in group 3 gave higher RADIANT scores to those vignettes in which they would be surprised if the patients were still alive after 12 months. In all groups, vignette B had the highest mean RADIANT score, followed by vignettes A and C, and the lowest on vignette D. Seventy-one percent of GPs in groups 2 and 3 considered SQ1 a helpful tool, and 75% considered SQ2 helpful.

**Conclusions:**

This innovative pilot study indicates that the majority of GPs think SQ2 is a helpful additional tool. The combination of the two “Surprise Questions” encourages GPs to make more specific plans for anticipatory palliative care.

**Electronic supplementary material:**

The online version of this article (10.1186/s12904-018-0312-6) contains supplementary material, which is available to authorized users.

## Background

Today most people in the Western world die of chronic, degenerative illnesses and malignancies, rather than of acute lethal diseases [1]. This implies that patients have a longer deteriorating trajectory, with increased symptom burden. For patients, carers, and family members, multidimensional supportive or palliative care is needed, preferably starting during disease-targeted care [1, 2]. The WHO defines palliative care as an early, multidimensional, proactive approach to maintaining or retaining quality of life alongside acceptance of mortality and bereavement. However, a means of demonstrating that a medical professional has fulfilled each of these requirements is not currently available [3].

Temel et al. demonstrated that timely palliative care in patients with metastatic non-small cell lung cancer alongside standard treatment increased their quality of life, reduced depressed mood, and even prolonged survival, as compared with standard treatment alone. Bakitas et al. showed that early initiation of palliative care as compared to late initiation led to an increase in life expectancy of cancer patients [4, 5]. Patients with other life-limiting diseases are also in need of early palliative care and may benefit from it [6–8].

Most patients in an advanced stage of a life-limiting disease live at home in the Netherlands, where the general practitioner (GP) acts as a gatekeeper and coordinator of caregiving. This makes the GP the appropriate health care professional to coordinate palliative care [9]. Unfortunately, despite efforts to promote timely palliative care planning and development of tools to identify patients who might benefit from this approach, most GPs only provide terminal, reactive care, mostly to patients with cancer [10]. Consequently, in many patients in an advanced stage of a life-limiting disease, anticipation of future problems, needs, the patient’s wishes, and mortality scenarios, does not occur in a timely fashion [3]. One of the barriers to providing anticipatory palliative care is identifying the right moment to start it, particularly in patients with organ failure or frailty [11]. To assist GPs in the identification of patients in need of anticipatory care planning, several tools have been developed [12]. However, most tools contain many items and are time-consuming to apply, when time is at a premium [13–15].

A way to assess the right moment to start palliative care that costs little time is the “Surprise Question” (SQ): *“Would I be surprised if this patient were to die in the next 12 months?”* [16] It appears to be sensitive for predicting 1-year mortality [17–23]. However, it has a low specificity and inadequate predictive value, which means that many identified patients live longer than one year. Therefore, just asking the SQ will identify a patient group that might be too large for a GP to provide structured, anticipatory care to. For that reason, there is a need for an easily applied tool with a higher specificity. Hence, we suggested an additional SQ that reads “*Would I be surprised if this patient is still alive after 12 months?”* when SQ1 is answered in the negative. Up to now, the value of the addition of this second SQ in encouraging proactive palliative care has not been explored, which makes this paper a unique contribution to the international literature.

We pilot tested whether adding SQ2 to SQ1 increases the thoroughness of palliative care planning among GPs and asked whether GPs think the double SQ is useful and applicable in daily practice.

## Methods

### Study design and participants

Between April and July 2016, we invited a random sample of 140 GPs in the south-eastern part of the Netherlands to participate in our pilot vignette study. Because the second SQ had never been studied before, we performed a pilot RCT. We estimated that about 30% of invited GPs would be willing to participate. Thus, by inviting 140 GPs to participate, we would have about 10 participating GPs per group. This number was expected to be sufficient to answer the research questions, keeping in mind the explorative character of this innovate study. In order to prevent socially desirable answering, we told participating GPs that the purpose of the study was to explore the influence of the type of patient illness on GP’s care planning, without mentioning that we were also interested in the influence of the SQs. For the same reason, we did not mention that they would be randomized. Forty-three GPs provided written informed consent, and were randomized to one of the four arms, after stratification for being a “specialized GP” (a GP who has completed an extensive additional course in Palliative care or in Elderly care). In each group, the GPs received a questionnaire with four vignettes [Additional file 1]. Group 1 was used as a control group. Group 2 received only SQ1 after each vignette and in group 3 both SQs (SQ1 and SQ2) were asked after each vignette.

Group 4 received an aid for anticipatory, multidimensional care planning after each vignette in addition to both SQs. However, this concerned another research question; for that reason, in this paper we will only report the results of groups 1–3.

### Procedures

All participating GPs received the randomization-assigned questionnaire online through Castor EDC (a valid research database software program), or by mail, according to their preference.

Each questionnaire contained four vignettes representing four different patient cases. Vignette A portrays a patient at an advanced stage of a chronic condition (chronic heart failure and chronic obstructive pulmonary disease), vignette B a patient with terminal metastatic pancreatic cancer, vignette C a frail lady with dementia and vignette D a relatively young patient with a recently diagnosed metastatic colon carcinoma. These vignettes were inspired by real anonymized cases from one of the authors (CV). [Additional file 1].

With each vignette, the respondent was asked if he would plan any kind of care for this particular patient. If so, they were asked to describe what instructions they would give to their young and inexperienced GP-trainee (who therefore needs explicit instructions) and about what kind of care needs to be planned for this patient in the upcoming period. At the end of the questionnaire, GPs in the corresponding groups were asked their opinions about the applicability and usefulness of, respectively, SQ1 and SQ2 [Table 1].

**Table 1:** Questions asked to the GPs regarding palliative care provision

1. Which aspects trigger you in general to start palliative care? (groups 1-3)* (2missing)
2. Do you think the Surprise Question, ‘Would you be surprised if this patient were to die within 12 months? ‘ would be helpful to identify patients in need of palliative care? (group 2)* (1 missing)
3. Is this Surprise Question applicable in daily practice? What are your concerns /barriers to apply them? (group 2)* (1 missing)
4. Do you think the second Surprise Question, in addition to the first SQ, would be of use in identifying patients in need of palliative care? (group 3)* (1 missing)
5. Are these two Surprise Questions applicable in daily practice? What are your concerns/barriers to apply them? (group 3) (1 missing)

*study group(s) that received this question

### Outcome measures

All invited GPs were asked to provide socio-demographics and palliative care experience-related data [Table 2]. All information provided in the questionnaire was collected anonymously in Castor EDC and blindly evaluated. As a scoring instrument did not exist to assess whether all requirements of the WHO palliative care definition are fulfilled, we created a scoring instrument in order to analyze the open text fields and quantify them, dubbed the RADboud Indicators of ANTicipatory care (RADIANT) score [Table 3]. Therefore, we searched for guidelines from Pallialine (a Dutch website with an evidence and consensus based collection of palliative care guidelines, powered by the Netherlands Comprehensive Cancer Organization; IKNL), requirements from the WHO concerning Palliative Care and information from thuisarts.nl (a Dutch website for patients, powered by the Dutch College of General Practitioners, with clear information about various subjects including treatment limitations and end-of-life preferences) and a list of discussion points about patients’ end of life, powered by the Royal Dutch Medical Association [3, 24–26].

**Table 2:** Baseline characteristics

Characteristic	Group 1	Group 2	Group 3	Non participants^a^
N	9	10	9	42^c^
Age mean	48	45	53	59
Male gender n (%)	5 (56)	3 (30)	6 (67)	19 (45)
Type of practice n (%)				
Single-handed practice	1 (11)	0 (0)	3 (33)	4 (10)
Two-man practice	3 (33)	6 (60)	2 (22)	12 (29)
Group practice	5 (56)	4 (40)	4 (44)	26 (62)
Years of working experience as GP, mean	16.7	14.5	22.5	20.5
GP trainer n (%)	2 (22)	6 (60)	6 (67)	15 (36)
Specialised GP^b^ n (%)	0 (0)	1 (10)	0 (0)	1 (2)
Estimated number of palliative patients / year, mean	5.1	6.4	6.7	5.7
Interest in palliative care (0: not at all; 10 extremely interested), mean	7.9	8.3	7.9	8.0
Self-assessed expertise in palliative care (0: no expertise; 10 extremely high expertise), mean	6.7	7.1	7.7	7.3

^a^GPs who were invited to participate in the study, but did not give informed consent (97) or gave informed consent but did not complete the vignette questionnaire (5)

^b^A GP that has followed an extensive additional course in palliative care or in elderly care

^c^GPs who provided socio-demographics and palliative care related data

**Table 3:** RADIANT scoring table

Item	Score	Provenance^a^
1. Discusses patient’s personal aspects of quality of life	1	x, y
2. Discusses how to achieve patient’s personal goals	1	q, x, y
3. Adheres to the patient’s preferences	1	q, y
Provides attention to the following dimensions:		q, x, y
4. Somatic problems		
5. Social context and finances		
6. Caregiving and activities of daily living		
7. Existential and psychological issues		
8. Involves other disciplines, including consultation team palliative care	1	y
9. Provides palliative care alongside disease-oriented care	1	x
Discusses advance care planning aspects:		
10. Hospital admissions	1	q, z
11. Antibiotics use	1	q, z
12. CPR policy	1	q, y, z
13. Mechanical ventilation	1	q, z
14. Treatment limitations – not specified	1	q, y
15. Dying scenario’s	1	q, y
16. Life prolonging treatments (e.g. artificial feeding and i.v. fluids)	1	q, z
17. Preferences for end-of-life care (e.g. palliative sedation, euthanasia)	1	q, y, z
18. Preferred place of death	1	q, y, z
19. Writes assignment for the out-of-hours GP cooperative	1	q
20. Involves family and loved-ones in care planning	1	q, x, y
21. Provides care to family and loved-ones	1	x, y
Maximum score	21	

^a^provenance, q Royal Dutch Medical Association [26], x WHO definition of palliative care [3], y Pallialine [24], z Thuisarts [25]

The primary outcome was the correlation between answer combinations for SQ1 and SQ2 (“Yes/No I would / would not be surprised if this patient were to die in the next 12 months” and “Yes/No I would / would not be surprised if this patient is still alive after 12 months”) and RADIANT scores. If an answer combination of SQ1: “No” and SQ2: “Yes” correlates with higher RADIANT scores than answer combination of SQ1: “No” and SQ2: “No”, it suggests that this answer combination prompts GPs to plan anticipatory care more thoroughly.

### Secondary outcomes were

*a.* differences in mean RADIANT scores per vignette A-D between the three GP-groups, particularly between the group with only SQ1 (group 2) and the group with both SQs (group 3). This difference in scores represents the extra value of the double SQ, as compared to SQ1 alone, in prompting GPs to plan anticipatory palliative care and the quality and thoroughness of care they described.

*b.* differences in mean RADIANT scores of all groups together between the cases in vignettes A-D. (Do the cases (type and progression of disease) influence the time-point, kind, and extent of care planning?)

*c.* differences between study group 1–3 in how often various aspects of the RADIANT score are mentioned in care plans.

*d.* Finally, with open questions, we qualitatively explored how GPs evaluated the single SQ and the double SQ.

### Statistical analysis

Continuous, normally distributed data were expressed as means and percentages. Because of the small numbers, no effect sizes were calculated.

### Ethical justification

The study was approved by the research ethics committee of Radboud university medical center in accordance with the Medical Research Involving Human Subjects Acts (WMO), case number 2016–2716. We obtained written consent from all participants to report individual anonymized data.

## Results

### Study participants

**Fig. 1:**
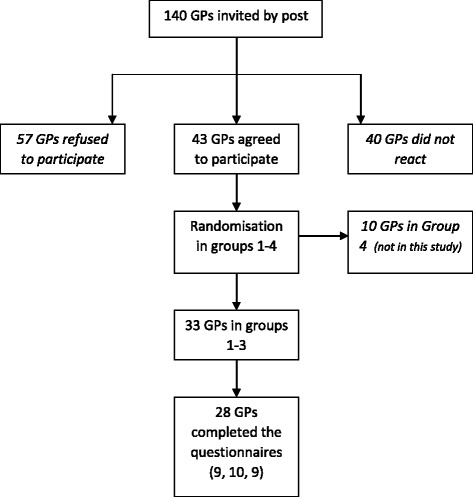
Flowchart study participants. Legend: -

Of the 140 GPs approached, 57 did not want to participate and 40 never answered our invitation; thus in total 97 GPs did not provide informed consent [Fig. 1]. Fourty-three GPs agreed to participate and were randomized into groups 1–4 and sent the corresponding questionnaire. Of those 33 were in groups 1–3. Twenty-eight GPs completed the questionnaire and 5 did not, mostly due to high workload or personal circumstances [Fig. 1]. Of these 28 GPs, nine were in group 1, ten in group 2, nine in group 3. Their characteristics are included in Table 2.

Characteristics of participants in the different groups were mostly similar. However, the percentage of males in group 2 was lower and the percentage of GP-trainers in group 1 was lower. The mean age was 49 (SD 9.2) and they had an average GP work experience of 18 (SD 9.4) years. Four GPs worked in an individual practice (14%), 11 GPs (39%) in a two-man practice and 13 (46%) in a group practice. Only one of them (4%) completed an additional course in palliative care or in elderly care and 14 (50%) were GP-trainers. Participants treated a mean of six palliative patients a year, and GPs rated their interest in palliative care as an 8.0 (scale 0–10; 0: not at all-10: extremely interested) and their self-assessed expertise in palliative care as a 7.2 (scale 0–10; 0: no expertise-10: extremely high expertise).

### Primary outcome measure: Correlation between answer combinations on SQ1 and SQ2 and RADIANT scores

In group 3, GPs who answered that they would not be surprised if this patient were to die in 12 months, and would be surprised if this patient is still alive after 12 months, had higher RADIANT scores than GPs in the same group who answered that they would not be surprised if this patient were to die in 12 months but they would also not be surprised if this patient is still alive after 12 months [Table 4].

**Table 4:** Answers on SQ1^a^ and SQ2^b^ and RADIANT scores^c^ per group^d^ and per vignette

Vignette	Group 2^e^ *n* = 10	RADIANT score group 2 mean	Group 3^f^ *n* = 9	RADIANT score group 3 mean
A^g^	10 SQ1 ‘no’	6.0	5 SQ1 ‘no’ & SQ2 ‘yes’	5.0
			4 SQ1 ‘no’ & SQ2 ‘no’	1.5
B^h^	10 SQ1 ‘no’	5.8	9 SQ1 ‘no’ & SQ2 ‘yes’	5.0
C^i^	9 SQ1 ‘no’	4.0	5 SQ1 ‘no’ & SQ2 ‘yes’	4.6
	1 missing		3 SQ1 ‘no’ & SQ2 ‘no’	4.0
			1 missing	
D^j^	5 SQ1 ‘no’	3.0	2 SQ1 ‘no’ & SQ2 ‘yes’	3.0
	4 SQ1 ‘yes’	1.8	3 SQ1 ‘no’ & SQ2 ‘no’	2.3
			3 SQ1 ‘yes’ & SQ2 ‘no’	0
			1 missing	

^a^SQ1: Would I be surprised if this patient were to die in the next 12 months?

^b^SQ2: Would I be surprised if this patient is still alive after 12 months?

^c^RADIANT score: see Table 3

^d^ only group 2 and group 3 are represented here, as group 1 did not receive any SQ

^e^group 2: received only SQ1 after each vignette

^f^group 3: received SQ1 and SQ2 after each vignette

^g^vignette A: advanced organ failure

^h^vignette B: end-stage metastatic pancreatic cancer

^i^vignette C: frailty

^j^vignette D: recently diagnosed metastatic colon cancer

### Secondary outcome measures

We did not find differences between GPs groups 1–3 in Radiant scores. Regarding vignettes A, B and D, group 3 has lower scores than group 1 and 2 [Table 5]. In all groups, vignette B (end-stage metastatic pancreatic cancer) had the highest mean score, followed by vignettes A (advanced organ failure) and C (frailty), and the lowest on vignette D (recently diagnosed metastatic colon carcinoma). In group 3, those vignettes of whom the GPs would be surprised if they were still alive after one year had mostly higher Radiant scores than the others. [Table 5].

**Table 5:** Mean cumulative RADIANT scores^a^ per group, per vignette and per SQ answer combination

Vignette	Group 1 n = 9	Group 2 n=10^b^		Group 3 n=9^b^		Mean *n* = 28
		SQ1^c^ all answers	SQ1 ‘no’	SQ1 all answers	SQ1 ‘no’ & SQ2^d^ ‘yes’	
A^e^	4.6	6.0	6.0	3.4	5.0	4.7
B^f^	5.9	5.8	5.8	5.0	5.0	5.6
C^g^	4.6	4.0	4.0	4.4	4.6	4.3
D^h^	2.9	2.7	3.0	1.6	3.0	2.4
Mean	4.5	4.6	4.7	3.6	4.4	

^a^RADIANT score: see Table 3

^b^some missing data: in both group 2 and group 3 one GP did not fill out vignettes C and D

^c^SQ1: Would I be surprised if this patient were to die in the next 12 months?

^d^SQ2: Would I be surprised if this patient is still alive after 12 months?

^e^vignette A: advanced organ failure

^f^vignette B: end-stage metastatic pancreatic cancer

^g^vignette C: frailty

^h^vignette D: recently diagnosed metastatic colon cancer

GPs mentioned specific treatment limitations least often in their care plans and in only three care plans (3%) GPs mentioned that they would discuss how to achieve patients’ personal goals. There were no striking differences between groups 1–3, except for involving other disciplines (50% of the case vignettes in group 1, compared to 20% for groups 2 and 3) and involving family and loved ones in planning care (36% of the case vignettes in group 1, compared to 22% and 25% in groups 2–3 respectively).

### GPs’ opinion about triggers to start palliative care

Many GPs mentioned that, in general, the diagnosis of a disease without curative options prompts them to start a palliative care approach. Their intuition, or information from a medical specialist, that death is approaching are considered important triggers. They also mentioned that they are prompted by frailty, complex situations, or when a patient suddenly or gradually has increased care needs. These latter situations give a GP the feeling of lagging behind events and of no longer being able to act proactively: *“*the feeling of being overtaken by events*.”* Also, the absence of a family caregiver or lack of a social network prompts them to initiate palliative care. Some GPs especially want to adhere to the patient’s wishes and start palliative care when the patient himself asks for it or gives other signals of being open to a conversation about palliative care: *“*the tangibility of the finiteness of life and the patient’s capacity to feel this too*.”* One GP in group 1 described SQ1 as an important trigger, another GP in group 1 mentioned SQ2, although they were not offered these questions and SQ2 has not been described before.

In the groups that received it, 12 out of 17 GPs who answered SQ1 (71%) considered it a helpful tool. However, GPs mentioned the inadequate specificity: “for anyone over 85, this could be their last year, but I will not start a palliative approach for all of them*.”* Regarding SQ2, 6 out of 8 GPs who answered it (75%) considered it helpful. Some of them already were familiar with SQ1 and found it helpful to determine the right moment to start anticipatory palliative care planning. One GP in group 3 noted: “I use the first surprise question on a regular basis, but the second one is new to me. They seem to be the same, but surely there is a nuanced difference. When the answer to SQ1 is ‘No’ and to SQ2 is ‘Yes’, it is clearer to me that the palliative phase has begun. The only objection is that these questions are subjective and therefore a physician can make a very inaccurate estimation. So I suggest answering these two SQs over again when the situation changes.”

## Discussion

Up to now, the classical SQ(1) alone was used and has proven to have low specificity as a tool to predict death, which may lead to a large and unselected group of patients for whom a GP should plan multidimensional, proactive, palliative care. Some participating GPs in this study underlined this shortcoming. Furthermore, its performance is insufficient in non-cancer patients [23]. Adding SQ2 to SQ1 may improve specificity as prognostic tool, but more meaningfully, combining the two SQs will help a medical professional to select a smaller, more accessible group of patients in need of anticipatory palliative care. The GP can then focus on these patients and plan palliative care more thoroughly.

Many tools have recently been developed to identify patients in need of palliative care in general practice, e.g. RADPACT, SPICT, NEPCAL, GSF [12]. These tools require more time investment and availability of indicators and are therefore less suitable for screening of patients in a GP’s daily practice. Also, most tools are focused on the palliative patient in the terminal phase, which implies that after identification there will be less time for proactive and anticipatory care planning. However, all of these tools are useful for meeting patients’ wishes, needs, and possible future scenarios and should be used in combination during conversations with the patient.

We did not study the prognostic value of the SQs. Already in 2005 Lynn suggested using the SQ to identify “whether the person is in a fragile enough condition that relatively minor worsening or intercurrent illnesses could lead to mortality. Some of the patients identified by the surprise question will end up living for years in a fragile state, and some will die soon, but all typically need the services that are priorities in the last phase of life: advance care planning, comfort measures, assistance for daily activities, family support, and so forth” [27]. Also Downar et al. noted the importance of using the SQ as a screening test for patients who might benefit from a palliative care approach [28]. Recognizing the patient in need is a prerequisite to starting proactive palliative care [29], not the prognosis.

Most participating GPs considered the already-known SQ1 and also the new SQ2 helpful.

As one of the triggers to start palliative care, some GPs mentioned “if a patient himself asks for it”. However, De Vleminck et al. demonstrated that patients, and particularly patients with disease trajectories other than cancer, are very unlikely to start discussing anticipatory care planning on their own initiative [30].

### Strengths and limitations

This is the first study in which the SQ is used in combination with case vignettes to see whether it prompts GPs to thoroughly plan proactive palliative care, instead of studying its prognostic value, which has been questioned before [31]. Adding a more specific trigger, SQ2, is also innovative.

However, the study also has several limitations. In this pilot study, the number of included GPs was limited and not sufficient to discover statistical significance. It was meant as a first exploration and therefore all results should be interpreted with caution. Although we aimed to use the results for a full-scale study, the relatively small percentage of GPs that wanted to participate in this vignette study led us to abandon this plan. Instead, we have started a prospective study in which GPs screened their patient list with both SQs. One year later, a retrospective medical record review will take place to determine sensitivity and specificity of both SQs and whether the different answers to these SQs are related to anticipatory care planning and care needs.

The response rate of 22% is about the same as in Schnakenberg et al.’s study (27.5%). In that study, GPs in Germany and Sweden were sent surveys about palliative care, which took no more than ten minutes to fill out [32]. Although we consider 22% fairly low, given the time required to complete the questionnaire (30–40 min) and Dutch GPs’ high workload, it is satisfactory.

Vignette 4, concerning the patient with recently diagnosed metastatic colon carcinoma, was not completed by two GPs and also had the lowest RADIANT score. We think that mainly the case itself will have influenced the score, but maybe the order in which the vignettes were presented might have influenced this outcome as well.

Although the RADIANT score is not a validated tool, it enabled us to quantify the open text fields and thus to compare groups not only qualitatively but also quantitatively. We chose anticipatory care to have a high share of the total RADIANT score, because we believe a medical professional can only provide good palliative care when, in addition to addressing actual problems, he also anticipates possible future problems. Besides, a medical professional can only be proactive when he is made aware of his patient’s condition, which is what we aimed to achieve with the SQs.

Whether the RADIANT score is related to the quality of a palliative care plan (the obtained scores are a fraction of the total possible score per vignette of 21 points) still needs to be studied in real patient care, as well as the validity and specificity of the tool.

Finally, we do not know whether all GPs in group 3 have understood the subtle but essential difference between SQ1 and SQ2. After all, we did not provide any explanation about the intention or use of this new SQ2.

### Recommendations

This simple, additional SQ2 was considered helpful by GPs and showed promising pilot results. In four prospective studies in, respectively, general practice, a medical oncology outpatient clinic, the Intensive Care Unit and nursing homes, we will further explore its usefulness and validity.

## Conclusions

In our pilot study, SQ2 in addition to SQ1 seems to contribute to more extensive and anticipatory palliative care planning for those patients of whom GPs would be surprised if they were still alive after 12 months, and to less extensive and anticipatory care planning when they would not be surprised if a patient were still alive after 12 months. Most GPs who received it considered SQ2 a useful addition to SQ1.

## Additional file

Additional file 1: Case vignettes. Case vignettes. Case vignettes, representing four different patient cases, inspired by real anonymized cases of one of the authors CV. (PDF 193 kb)

## Abbreviations

GP: General Practitioner
RADIANT: Radboud Indicators of Anticipatory care
SQ1: Would I be surprised if this patient were to die within the next 12 months?
SQ2: Would I be surprised if this patient is still alive after 12 months?
