# Supplementary material for: Adding a second surprise question triggers general practitioners to increase the thoroughness of palliative care planning: results of a pilot RCT with cage vignettes
Source: BMC Palliat Care. 2018 Apr 19;17:64. doi: 10.1186/s12904-018-0312-6 (PMC5909217; doi:10.1186/s12904-018-0312-6)
Supplement: Supplementary file 1 — Case vignettes. Case vignettes. Case vignettes, representing four different patient cases, inspired by real anonymized cases of one of the authors CV. (PDF 193 kb) [file 12904_2018_312_MOESM1_ESM.pdf]

Additional file 1: Case vignettes

Vignette A

Mrs A., 83 years old, is widowed and lives independently in a detached house on the outskirts of the village. She and her husband used to run several shops in the centre of the nearest city. She has six children, who are all closely involved. She enjoys life, and particularly the company of her children and grandchildren.

Her cognitive functions are still excellent, but she has several relevant somatic problems, the most important of which are:

COPD Gold III-IV. She still enjoys several cigarettes per day

Presbyacusis, for which she wears bilateral hearing aids

Diabetes type 2

Kidney failure (MDRD < 30)

Anaemia eci

In 2011, she suffered a severe myocardial infarction, for which she underwent an emergency PTCA and coronary stent placement. Unfortunately, she developed severe heart failure (NYHA classification 3-4).

Her main complaints are fatigue and some exertional dyspnoea. Her exercise capacity is clearly decreasing and walking longer distances within her house is sometimes challenging. In the past year she has experienced several acute exacerbations of both heart failure and COPD, often combined. She regularly asks you about treatment options regarding her fatigue, since she still very much enjoys life and does not want to say farewell to her children yet.

Vignette B

56-year-old Mr W., is married and lives with his wife in an apartment on the edge of the forest. His wife is 52 years of age and very healthy. They have two daughters and five grandchildren. Both daughters are involved and live in the same area.

Mr W. is a manager at the university. He is rarely ill but since a few months he has been having complaints of his upper abdomen. Apart from a fluctuating but sometimes severe pain, he experiences general malaise and an overall decline in his physical abilities. His condition was difficult to diagnose at first, but eventually a metastatic pancreatic tumour was found. Currently, he is suffering severe pain and he has lost several kilograms of weight. A coeliac plexus blockade has been planned. He is only moderately fit, but calm and resigned.

Vignette C

Mrs C. is a 91-year old widow. She lives alone in a luxurious service apartment (an apartment where additional care is provided). She has no children; just a sister in law (who is 85+ herself), who cares for her.

Lately, Mrs C. has been experiencing increasing problems in her daily life: she hardly leaves her house and certain complex tasks tend to fail. Her (short-term) memory seems to be intact. She is, however, increasingly disoriented in time and place (orientation in person is intact). A year and a half ago she was diagnosed with dementia by a geriatrician.

Several years ago, she had a heart attack, for which she was hospitalized. She was eventually treated conservatively with medication. Next, she developed heart failure, which is currently stable and inactive. She also has atrial fibrillation, for which she takes oral anticoagulants.

She also developed squamous cell skin cancer in her face several times. Recently, one of those cancers, situated at the right side of her mount, was surgically removed. Because of postoperative complications, she had to undergo a repeat surgery. This resulted in permanent dysfunction of the right side of her mouth which causes eating difficulties. She lost several kilograms of weight in the past months.

Finally, she suffers from bilateral cox arthrosis, which causes pain every now and then.

Lately Mrs C. has fallen regularly. Since her last fall she has been immobilized due to severe lower back pain. She spends the majority of the day either dozing in her chair or lying in bed. Two years ago she went through an episode were she experienced the same problems, at that time caused by osteoporotic vertebral infraction. She recovered spontaneously from this previous episode in 6 months.

Vignette D

69-year-old Mr T. is married and lives with his wife in a single-family home. He is quite healthy and hardly ever ill.

After a period of vague abdominal complaints, he was referred to an internist, who diagnosed him with colon cancer with liver and lung metastases. At first, Mr T. was very emotional about this news but after a while he regained his calm.

Mr T. would like to receive life-prolonging treatment. He is very fit and hardly experiences any complaints at the moment. Shortly he has an appointment with a medical oncologist to discuss the possible options. He is very motivated to continue treatment.
